# Human Pasteurellosis Health Risk for Elderly Persons Living with Companion Animals

**DOI:** 10.3201/eid2502.180641

**Published:** 2019-02

**Authors:** Sándor Körmöndi, Gabriella Terhes, Zoltán Pál, Endre Varga, Mária Harmati, Kriszina Buzás, Edit Urbán

**Affiliations:** University of Szeged, Szeged, Hungary (S. Körmöndi, G. Terhes, Z. Pál, E. Varga, K. Buzás, E. Urbán);; Hungarian Academy of Sciences, Szeged (M. Harmati, K. Buzás)

**Keywords:** infection, Pasteurella infections, pasteurellosis, elderly, companion animals, bites, animals, bacteria, Hungary

## Abstract

The necessity for diagnosis and treatment of this infection is emphasized by the high number of complications and death rate.

*Pasteurella* spp. infections can cause various diseases in wild and domestic animals; in humans, most infections are associated with cat or dog bites, licks, and scratches (*1*). Annually, in the United States, ≈300,000 visits to emergency departments for animal bites or scratch wounds are recorded; however, not all of these are associated with infections. Regarding animal bites, 3%–18% of dog bites and 28%–80% of cat bites become infected (*2**,**3*); 50% of dog bites and 75% of cat bites are associated with the presence of *Pasteurella multocida* (*3*), which can be frequently detected as part of the oral microbiota in various animals such as cats, dogs, pigs, and various wild animals (*2**,**3*). 

Data about injuries associated with animal bites or scratches in Hungary are not available; however, rabies vaccines are given to >4,000 patients annually (4,784 in 2017) (*4*). This number represents mainly cases in which the animal could not be observed after an accident or injury (*4*). Of the 35,000 traumatic injury cases (such as various injuries from accidents, bites, scratches, sport injuries, falls, and injuries that occur at home) that are recorded annually at our university hospital in Szeged, Hungary, 14–37 (0.04%–0.1%) are associated with animal bites or scratches.

Localized infections caused by *Pasteurella* spp. are characterized by cutaneous inflammation that usually develops shortly after animal bites or scratches. Later, local complications including osteomyelitis, septic arthritis, and abscess formation or systemic infection such as infection of large articulated joints, meningitis, intraabdominal infection, sepsis, and pneumonia may develop (*5*). In immunocompromised patients, the infection may manifest as severe pneumonia, sepsis, or a fatal form of pasteurellosis. In patients with underlying pulmonary disease, pneumonia, empyema, and lung abscess caused by *Pasteurella* spp. can be detected, whereas in patients with liver dysfunction, sepsis has been described (*3*). Other severe invasive infections (meningitis, endocarditis, and peritonitis) caused by various *Pasteurella* spp. have also been described; however, their incidences are rare (*6*)*.*

During the past 10 years, we have observed that the rate of human pasteurellosis is rising in Hungary, and in several cases of invasive pasteurellosis, animal contacts are usually not mentioned. In the literature, most papers dealing with human pasteurellosis are reports of individual cases; only a few papers have reported on all cases recognized in a hospital. To achieve better knowledge of human pasteurellosis, we collected data about both localized and invasive forms of human infections and compared the results with international data.

## Methods

### Study Design and Patients

We included patients in this study if they had microbiological investigation with positive results for *Pasteurella* spp. in the local university hospitals in Szeged, Hungary (intensive care unit and departments of traumatology, surgery, pediatrics, dermatology, ophthalmology, obstetrics and gynecology, otorhinolaryngology, and head and neck surgery) during 2002–2015. For culture, we used Columbia agar with 5% sheep’s blood and chocolate agar with PolyViteX (bioMérieux, https://www.biomerieux.com) and incubated the cultures at 37°C for 24 h in a 5% CO_2_ incubator. We performed identification using the VITEK 2 GN ID and API NH Systems (bioMérieux) before 2012; after 2012, we used matrix-assisted laser desorption/ionization time-of-flight (MALDI-TOF) mass spectrometry (Bruker Daltonik, https://www.bruker.com). We obtained patients’ medical history from the local medical database. We identified local pasteurellosis if the patient had a superficial wound or tenosynovitis or if small joints were involved. Invasive pasteurellosis was present if large joints, including shoulder, hip, knee, or prostheses, were inflamed or if the patient had neurologic, pulmonary, cardiovascular, abdominal, or pelvic involvements or had septic shock or bacteremia. 

This retrospective study was approved by the University Research Ethics Committee, Faculty of Medicine, University of Szeged, Hungary. Collection of data about participating patients was in accordance with ethical standards at the institutional and/or national research committee and with the 1964 Helsinki Declaration and its later amendments. 

### Data Analysis

We collected the following data from the medical database: age, sex, concurrent medical conditions, animal exposure history, outcomes, and laboratory findings at hospital admission. For statistical analysis, we collected and analyzed demographic and clinical data about the patients. We used descriptive statistics including means or medians with ranges and percentages to characterize data; in this case, we used Microsoft Excel 2013 (https://www.microsoft.com). We used Mann-Whitney U-test or χ^2^ tests with Yates’ correction to compare groups. A p value <0.05 was considered statistically significant. We analyzed the data using GraphPad Prism version 5.03 (https://www.graphpad.com/scientific-software/prism).

## Results

### Baseline Characteristics of Patients with *Pasteurella* spp. Infection

**Figure 1:**
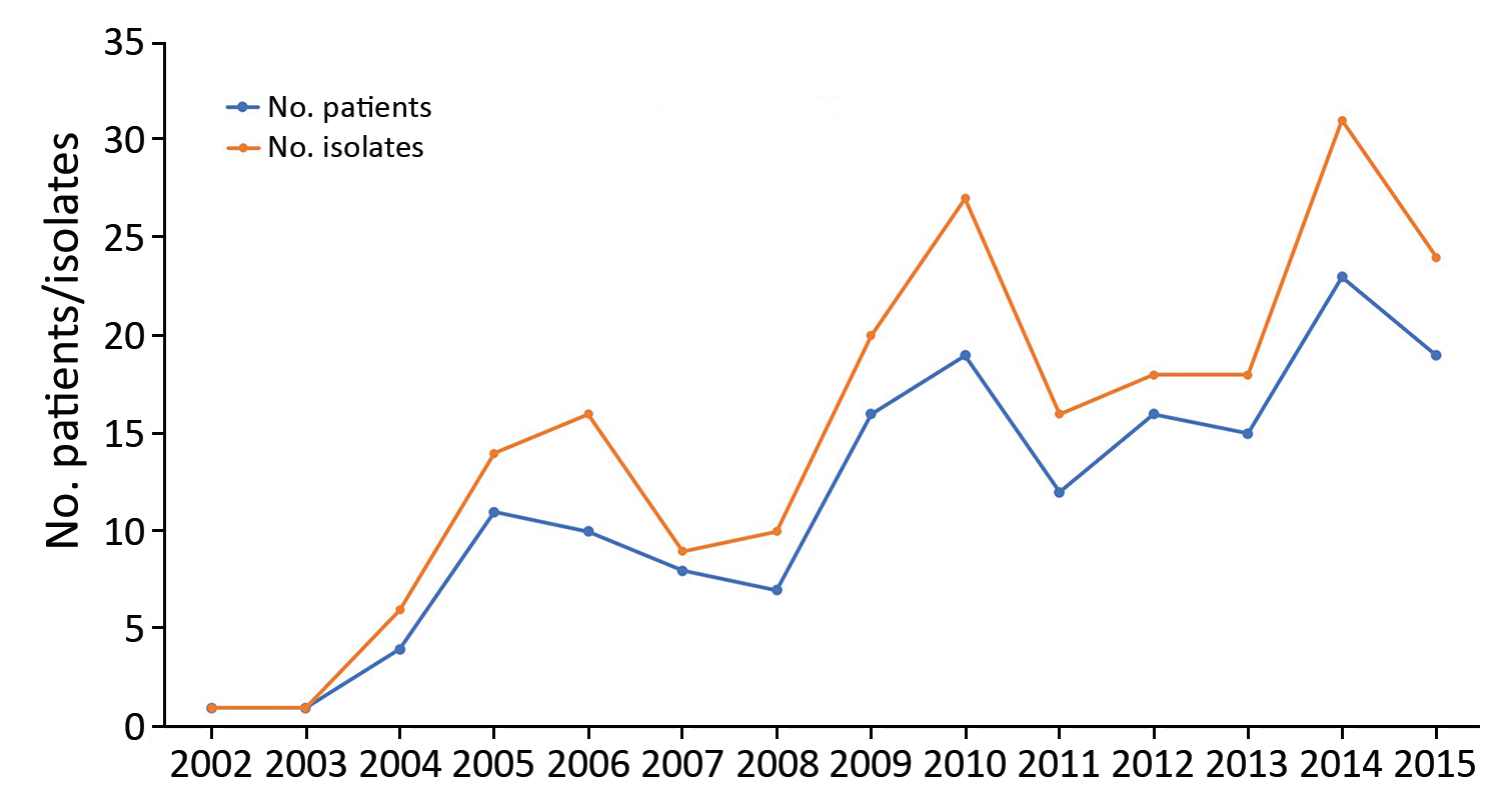
Increasing tendency in annual rates of human pasteurellosis and in the number of *Pasteurella* isolates, Szeged, Hungary, 2002–2015.

**Figure 2:**
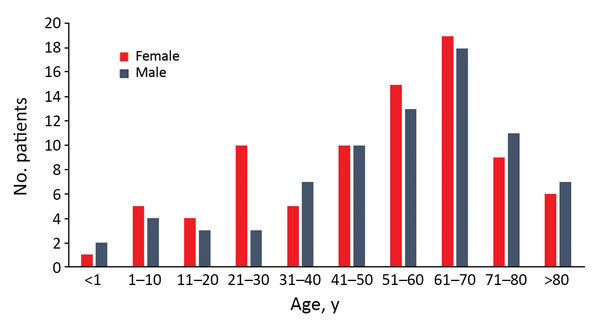
Distribution of pasteurellosis cases (N = 162) according to age group and sex, Szeged, Hungary, 2002–2015.

For 2002–2015, we isolated 211 *Pasteurella* spp. from 162 patients; we found that the number of isolates and human pasteurellosis cases has increased from year to year (Figure 1). The median age of patients with positive culture results was 57 (range 0–97) years. The proportion of *Pasteurella* spp. isolation was approximately equal for men (n = 78, 48.1%) and women (n = 84, 51.9%), and we observed increasing rates of pasteurellosis cases by advanced age in both sexes (Figure 2). 

We determined the distribution of *Pasteurella* spp. in clinical specimens (wounds, respiratory tract, and abdominal specimens; blood, synovial, cerebrospinal, and pleural fluids) (Table 1). Most strains (n = 155, 73.5%) were isolated from wound specimens (Table 2), but some strains originated from unusual sites such as synovial fluid, pleural fluids, and abdominal specimens. In the respiratory specimens (n = 23), 5 strains were isolated from the upper respiratory tract; among these, asymptomatic carriages were considered in 3 patients. Two *P. multocida* strains isolated from the upper respiratory tract were also present in pleural fluid and bronchoalveolar lavage of the same patient with severe respiratory failure. In lower respiratory specimens, invasive infections were documented when pure cultures of *Pasteurella* spp. and high numbers of granulocytes were detected and the patient had symptoms of lower respiratory tract infections. All strains (n = 18) isolated from the lower respiratory specimens were grouped into the invasive infection cluster.

**Table 1:** Distribution of *Pasteurella* spp. in clinical specimens from patients in Szeged, Hungary, 2002–2015

Species	No. (%) isolated strains
*P. multocida*	160 (75.8)
*P. canis*	36 (17.1)
*P. pneumotropica*	11 (5.2)
*P. dagmatis*	2 (0.9)
*P. stomatis*	1 (0.5)
*P. aerogenes*	1 (0.5)
Total	211 (100)

**Table 2:** Distribution of clinical specimens positive for *Pasteurella* spp. from patients in Szeged, Hungary, 2002–2015

Specimens	No. (%) isolates
Wound	155 (73.5)
Respiratory tract specimen	23 (10.9)
Blood	14 (6.6)
Synovial and pleural fluids	4 (1.9)
Abdominal	3 (1.4)
Cerebrospinal fluid	1 (0.5)
Others (middle ear, sinus, conjunctiva, dialysis catheter)	11 (5.2)
Total	211 (100)

### Polymicrobial *Pasteurella* sp. Infections

In 79 patients, *Pasteurella* sp. was the only isolate, whereas polymicrobial infections were detected in 83 patients (51.2%). In these cases, the most frequent anaerobic bacteria isolated from clinical specimens were *Fusobacterium nucleatum* (n = 18), *Peptostreptococcus anaerobius* (n = 11), *Prevotella oralis* (n = 10), *Prevotella melaninogenica* (n = 7), *Prevotella loescheii* (n = 7), and *Bacteroides pyogenes* (n = 7). Among facultative anaerobic bacteria, *Staphylococcus aureus* (n = 12) and *Escherichia coli* (n = 5) were the most common species.

### Characteristics of Localized and Invasive Pasteurellosis

Localized infections secondary to bites, scratches, or licking were the most prevalent type during the study period (n = 114, 70.4%). Most patients with localized infections were female (n = 68, 59.6%). For invasive infections (n = 48), however, male predominance (n = 32, 66.7%) was observed, and that difference was statistically significant (χ^2^ = 8.345, df = 1, p = 0.004). The difference in the age distribution among those with localized (median age 53.5 [range 0–97] years) and invasive (median age 63 [range 0–87] years) infection was also statistically significant (Mann-Whitney U = 2142; p = 0.029). In the invasive-infection group, the largest number of patients (n = 12, 25%) had various abscesses (e.g., liver abscess, pulmonary abscess, abscesses after sigmoid resection); 8 (16.7%) patients had pneumonia, and 8 (16.7%) had bacteremia. Respiratory failure was diagnosed in 6 (12.5%) patients, osteomyelitis was in 4 (8.3%) patients, pleurisy in 2 (4.2%) patients, and arthritis in 2 (4.2%) patients. Central nervous system infection, adnexitis associated with pelvic inflammatory disease, peritonitis, pacemaker infection, cirrhosis, and gangrene were also recorded.

In 8 (7%) patients with localized infections and 37 (77.1%) patients with invasive infections, underlying diseases were recorded in the medical history (Table 3); the difference between the 2 groups was statistically significant (χ^2^ = 72.59, df = 1, p<0.0001). Some patients with invasive infections had multiple underlying disorders. Cardiovascular disease, diabetes, and malignancy were the most frequent underlying diseases.

**Table 3:** Presence of underlying disease in cases of local and invasive *Pasteurella* spp. infections among patients in Szeged, Hungary, 2002–2015

Underlying disease	No. (%) patients	
	Local infection	Invasive infection
Malignancy	0	10 (20.8)
Cardiac	0	6 (12.5)
Diabetes mellitus	4 (3.5)	6 (12.5)
Pulmonary	0	2 (4.2)
Hepatic (cirrhosis)	0	2 (4.2)
Prosthesis	0	2 (4.2)
Psychiatric	1 (0.9)	1 (2.1)
Genetic (Down syndrome)	0	1 (2.1)
Multiple	1 (0.9)	7 (14.6)
No data	106 (93)	11 (22.9)
Total	114 (100)	48 (100)

Of 114 patients with localized infection, 94 (82.5%) had contact with a dog (n = 41, 43.6%) or cat (n = 53, 56.4%). Ten patients had no animal contact, and no data were available for 10 patients. Animal contacts were recorded in 10 (20.8%) of 48 cases of invasive infection; no information about animal contacts was available in 16 (33.3%) cases. Twenty-two (45.8%) of 48 patients with invasive pasteurellosis had no animal contact.

**Figure 3:**
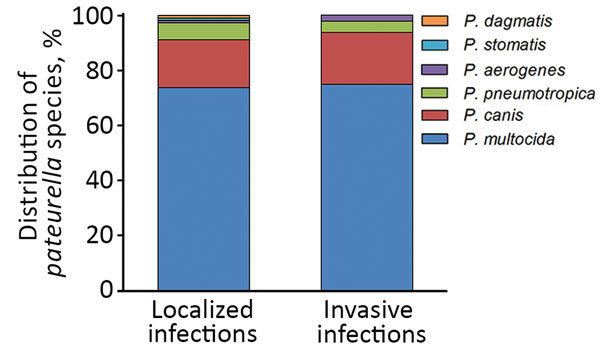
Distribution of various *Pasteurella* spp. in localized and invasive infections, Szeged, Hungary, 2002–2015.

Most injuries (74.6%) in the localized-infection group were attributed to animal bites. Scratch wounds caused by cats were recorded in 12 patients; however, some of them had simultaneous bite injuries. Six cases of cat-associated injury were observed in women 21–30 years of age; in the same age group of male patients (3 patients), none had animal contact (Figure 2). Differences in the distribution of various *Pasteurella* spp. between localized and invasive infections could not be detected (Figure 3).

When we compared data about hospitalization in patients with localized and invasive infections, we found 71 (62.3%) of 114 patients with localized infections had hospitalizations; the average length of stay was 8 (range 1–30) days. In the invasive-infection group, 40 of 48 patients (83.3%) were hospitalized, and the average length of hospital stay was 12.7 (range 1–60) days. Patients with invasive infections were more frequently hospitalized than patients with localized infections (χ^2^ = 5.999, df = 1, p = 0.014), and the average length of stay among patients with invasive pasteurellosis was also longer. Hospital admission was almost the same in patients with dog bites (68.3%) and in patients with cat-induced injury (64.2%). The average length of hospital stay was longer (11 [range 1–60] days) in cases of dog bites than in cases of cat bites or scratches (8.3 [range 3–41] days). In patients with localized infections, injuries affected mostly the upper extremities (75 patients, 65.8%); in 23 patients, lower extremities were affected, mainly shins; in 6 patients, the face; in 1 patient, the eye; and in 1 patient, the genitals.

### Complications after Localized and Invasive Pasteurellosis

Ileostomy, hysterectomy, skin transplantation, and amputation were documented in 4 cases of invasive pasteurellosis. Ileostomy was performed because of sudden onset of perforation; during the surgical procedure, a sample from the abdominal cavity was collected for microbiological culture, and *P. multocida* was isolated. Total abdominal hysterectomy and bilateral salpingo-oophorectomy were performed in a young woman with bilateral tubo-ovarian abscess due to *P. multocida* infection. A pacemaker electrode, a prosthesis, and a catheter were removed in 3 different cases because of endocarditis, recurrent inflammations around knee prosthesis, and purulent drainage around the dialysis catheter.

After localized *Pasteurella* sp. infection, severe complications were registered in 23 cases (20.2%); functional disability, mainly in fingers, developed in 10 patients (6 cases associated with dog bites, 4 cases with cat bites and scratches); and lymphangitis was observed in 7 of 53 (13.2%) patients. Following bites and scratches, 3 patients had amputations (2 from dog bites, 1 from a cat bite), affecting mainly the fingers; 3 patients had osteomyelitis (associated with dog bites) as a consequence of pasteurellosis. The high number of complications emphasizes the importance of localized and invasive pasteurella infections.

Of the 162 patients with pasteurellosis, 143 (88.2%) patients recovered; in 6 cases, no data were available about the outcome of pasteurellosis (in these cases, patients were transferred to other hospitals). Thirteen of the 162 patients died; all these patients had invasive pasteurellosis infections. Seven of these 13 patients had pneumonia and respiratory failure, 5 patients had bacteremia, and 1 patient had panencephalitis after traumatic brain injury. The median age of patients who died was 69.5 (range 39–84) years; most of them had multiple underlying diseases. Laboratory investigations revealed elevated leukocytes, C-reactive protein levels, and liver enzymes in all patients who died from pasteurellosis. In 4 cases, animal contacts, including dogs, cats, and other animals, were recorded in the patient’s medical history. No information about animal contact was available in 5 cases, and in 4 cases, no animal contact could be found. In cases of animal contacts, no bite or scratch was mentioned by the patients or relatives. 

From clinical specimens (respiratory tract specimens, cerebrospinal fluid, and blood), *P. multocida* was isolated from 12 patients, and *P. canis* from 1 patient. All 13 of these patients were hospitalized; the average of length of hospital stay was 10.3 (range 1–30) days. *Pasteurella* sp. was isolated from respiratory specimens or pleural fluid from 6 patients, from blood culture from 4 patients, from 2 wound specimens, and from 1 cerebrospinal fluid specimen.

## Discussion

According to data in the literature, two thirds of human infections are zoonotic in origin. Among these infections, human pasteurellosis is not a common cause of death in humans because of commonly used prophylactic treatment after animal bites or scratches; however, deaths caused by *Pasteurella* sp. infection have been increasing in the United States (*3*). We found that 162 patients had *Pasteurella* infections during 2002–2015 in our university hospital in Hungary. We found a higher number of cases caused by various *Pasteurella* species than similar surveys showed earlier, such as those by Giordano et al. (*7*) and Nollet et al. (*5*). To our knowledge, most publications dealing with human pasteurellosis showed only a few cases or reviewed only unusual infections caused by certain *Pasteurella* spp.; only 1 publication (*5*) presented data, from ≈102 patients, to determine risk factors for invasive pasteurellosis. Those surveys showed that higher mean age and underlying diseases, mainly chronic liver disease and neoplasia, are commonly associated with invasive pasteurellosis. Our data confirmed that invasive infections are more frequent in elderly patients and that these patients usually have 1 or more underlying diseases. 

Giordano et al. (*7*) and our results also showed that the detection of *Pasteurella* spp. from the blood or respiratory tract was frequently associated with the absence of animal bites; at the same time, skin infections were usually attributed to animal bites. Most hospitalized patients with animal bites were admitted because of cat bites. Patients without animal bites were more frequently hospitalized, and their length of stay was longer, compared with patients who had animal bites. Patients treated in the ICU did not have animal bites, and most of them had no animal contact, as well. In our study, among patients with invasive pasteurellosis, the death rate was 27.1%. Similarly, the death rate was 21% in Giordano et al.’s observational study (*7*); in Nollet’s study, the death rate from invasive infections was 11% and the death rate for localized infections was 1.4% (*5*).

The French Pasteurella National Center has reported that the prevalence of central nervous system infection caused by *Pasteurella* spp. is <1% of all *Pasteurella* infections (*8*). In adults, meningitis caused by *Pasteurella* infection is usually associated with cranial trauma or surgery or chronic otitis (*9*). Our data confirmed these findings. The prevalence of central nervous system infection caused by *Pasteurella* sp. was 0.6%. One patient with meningitis had a traumatic injury to his head from a car accident and was found near a stable; thus, the source of this infection was deemed to be the environment.

In the literature, dog bite is the most common cause of bite injury, followed by cat bite. Most bite injuries are minor wounds; thus, patients generally do not seek medical attention. Therefore, the reported prevalence of bite-related wounds is probably the tip of the iceberg. Dog bites may be associated with fractures because of their high energy, whereas cat bites frequently cause puncture wounds, leading to the development of tenosynovitis (*10*). Wounds caused by dog bites are less frequently infected than wounds caused by cat bites. Our investigations supported this observation, because patients with cat bites or scratches have pasteurellosis more frequently than do patients with dog bites. Literature data show that cat bites or scratches are more dangerous than dog bites because the injuries tend to be deep and are therefore difficult to clean properly (*2*). When we studied the complications following dog or cat bites, we observed that after dog bites, the rates of functional disability, osteomyelitis, and amputation were slightly higher than for those after cat bites. Lymphangitis could not be detected after dog bites; this finding was not described earlier.

*P. multocida* subsp. *multocida* is the most frequent clinical *Pasteurella* isolate in humans, ahead of *P. canis* and *P. multocida* subsp. *septica* (*11*). This fact was also confirmed by our investigations; 68.7% of *Pasteurella* strains were *P. multocida*, the second most common species was *P. canis* (17.1%), and third was *P. pneumotropica* (5.2%). Differences between species isolated from localized and invasive infections could be detected in our study, a finding also confirmed by Nollet et al. (*5*).

We followed the rates of human pasteurellosis and changes in the number of *Pasteurella* isolates from year to year, and we observed increasing tendencies in both groups. This finding may be explained by the increasing number of animals in households, and close intimate contact with pets. Approximately 57% of households in Canada own >1 companion animal, whereas only 39% of persons >65 years of age have a pet (*12*). Similar data are not available in Hungary, but according to unofficial data, >3 million dogs and ≈3 million cats live in Hungary (human population 9.938 million). In the Canada survey, the rate of pet ownership was the highest among middle age person (*12*), and pet-associated infections usually originated from injuries or animal bites; we saw a similar trend in our survey. In elderly patients, because of age-related dysfunction of the immune system or underlying disease, the risks for pet-associated disease and invasive infections are increased.

Another possible explanation for the increasing tendency in the rate of human pasteurellosis is the development of microbiological methods for identification, such as use of MALDI-TOF mass spectrometry and molecular methods. However, the possible role of these methods in explaining the higher number of isolates or human pasteurellosis cases could be excluded in study because we started to use MALDI-TOF mass spectrometry after 2012 but the increasing tendency in pasteurellosis could be observed before 2012. In addition, the spectrum of isolated various *Pasteurella* spp. did not change after the introduction of MALDI-TOF mass spectrometry and molecular methods; earlier automated identification systems provided adequate identification of *Pasteurella* strains.

The retrospective nature of this study and data interpretation have some limitations. Medical records for inpatients are completed better than those for outpatients; this may lead to an underestimate of concurrent conditions for outpatients, who fall mostly into the localized-infection group. In several cases, medical records did not contain information about animal contact (mainly in case of non–bite-associated pasteurellosis), which may also cause underestimation of the number of animal contacts. The number of infections caused by *Pasteurella* spp. is probably higher than we observed, because patients, mainly those in small villages, can receive emergency treatment from a local general practice physician.

Pets, including dogs and cats, are frequently recommended to patients with chronic illness because animal therapy might provide a potential health benefit. Elderly patients may also own companion animals to combat loneliness; however, pets may be the potential source of various infections or injuries (*13*). Our retrospective survey showed that the rate of human pasteurellosis over a 13-year study period in Hungary increased from year to year and with advanced age, and the number of *Pasteurella* infections was higher than it was in earlier studies. We observed that *Pasteurella* patients with cat- and dog-associated injuries were frequently hospitalized. In cases of invasive infections, the source of *Pasteurella* infection was frequently unknown. In spite of the adequate treatment on the basis of medical chart review and antimicrobial drug susceptibility of the isolated strain, the death rate from these infections was 27.1% in our study. We also found that complications after localized infections were detected frequently, and certain complications, such as lymphangitis, are associated only with injuries caused by cat bites; this connection was not described in earlier publications. We think that the rate of pasteurellosis is much higher than estimated because many patients with smaller injuries do not seek medical advice, and in many cases, general practice physicians try to treat smaller injuries and do not perform sample collection or order microbiological investigations. Our results, as well as international results, show that education about the possible health hazards associated with pet ownership should be provided, and the increased risks for infection in elderly and immunocompromised patients should be emphasized.
